# Comparison between haloperidol decanoate and oral haloperidol on seeking psychiatric emergency care

**DOI:** 10.1192/j.eurpsy.2022.1508

**Published:** 2022-09-01

**Authors:** L. Osaku, G. Salvadori, M. Porcu

**Affiliations:** Maringá State University, Medicine, Maringa, Brazil

**Keywords:** haloperidol decanoate, haloperidol, recurrence, Antipsychotics

## Abstract

**Introduction:**

Haloperidol is a first generation, high potency, low cost and widely used antipsychotic. There are inconsistencies in the literature about comparison of effectiveness between long-acting injectable haloperidol (HDLAI) with oral haloperidol (OH), as well as the combined use of both formulations (HDLAI+OH).

**Objectives:**

To verify whether HDLAI reduces the number of emergency visits and hospitalizations when compared to oral OH, or in combination therapy HDLAI+OH.

**Methods:**

Retrospective observational study on a Psychiatric Emergency department, including patients aged 18 to 60 years, both genders, under continuous treatment for at least 5 months with Haloperidol for any psychiatric illness, divided into 3 groups of patients (HDLAI, OH, HDLAI+OH). Dependent variables: visits and admissions. Independent variables: sex and age. Data were checked for normality (Kolmogorov-Smirnov test) and homoscedasticity (Bartlett test). For comparison of average number of visits and hospitalizations of patients Kruskal-Wallis test followed by Dunn’s multiple comparison test was used. It was considered statistically significant if p < 0.05. This study was approved by the Ethics Committee of Maringá State University.

**Results:**

No statistical difference between groups HDLAI and OH was found. The HDLAI+OH group presented higher means of emergency visits and hospitalizations with statistical significance.

**Conclusions:**

It suggests the use of HDLAI can be considered an alternative as effective as oral intake. Prolonged use of associated HDLAI and oral supplementation leads to worst outcomes.

**Disclosure:**

No significant relationships.

